# Right ventricular dysfunction for prediction of long‐term recovery in *de novo* HFrEF : a PROLONG‐II substudy

**DOI:** 10.1002/ehf2.15236

**Published:** 2025-02-04

**Authors:** Aiste Monika Jakstaite, Johanna Mueller‐Leisse, Henrike A.K. Hillmann, Stephan Hohmann, Jörg Eiringhaus, Udo Bavendiek, Tibor Kempf, Christian Veltmann, Johann Bauersachs, David Duncker, D. Berliner

**Affiliations:** ^1^ Department of Cardiology and Angiology Hannover Medical School Carl‐Neuberg‐Str. 1 Hannover Germany; ^2^ Electrophysiology Bremen Heart Center Bremen Senator‐Wessling‐Str. 1 Bremen Germany

**Keywords:** Right ventricular function, Heart failure, Speckle‐tracking echocardiography, Right ventricular free wall longitudinal strain, Right ventricular‐pulmonary arterial coupling

## Abstract

**Aims:**

To analyse the predictive value of advanced markers of right ventricular (RV) function and RV‐pulmonary arterial (PA) coupling in forecasting long‐term left ventricular (LV) improvement in *de novo* heart failure with reduced ejection fraction (HFrEF).

**Methods and results:**

260 patients (mean age 57 years, 68% men) from the PROLONG‐II study were included. PROLONG‐II analysed patients with new‐onset HFrEF receiving a wearable cardioverter‐defibrillator. For this substudy, RV free wall longitudinal strain (RVFWS), tricuspid annular plane systolic excursion (TAPSE), fractional area change (FAC), and right ventricular‐pulmonary artery (RV‐PA) coupling ratios [RVFWS/systolic pulmonary artery pressure (PASP), TAPSE/PASP and FAC/PASP] at baseline and 3‐month follow‐up (early follow‐up) were examined. LV improvement and non‐improvement were defined as an LV ejection fraction (LVEF) of >35% or ≤35% at last available (long‐term) follow‐up. The median follow‐up was 31.5 months (IQR: 18.2–45.4), and 151 (58%) patients experienced LV improvement in the long term. No significant differences of RV function and markers of RV‐PA coupling were observed at baseline; however, the subgroup of patients with long‐term LVEF improvement showed better RV function at early follow‐up (RVFWS −20.9 ± 4.3 vs. −18.5 ± 5.1%, TAPSE 19.7 ± 5.1 vs. 17.4 ± 4.9 mm, FAC 39.7 ± 8.5 vs. 35.2 ± 9.4%, all *P* < 0.01). In multivariable analysis, RVFWS at early follow‐up was shown to be an independent predictor of later LV recovery [odds ratio 1.078 (95% confidence interval 1.010–1.150), *P* < 0.05]. The non‐improvers exhibited worse RV‐PA coupling at early follow‐up [RVFWS/PASP 0.82 ± 0.35 vs. 0.65 ± 0.35%/mmHg, TAPSE/PASP 0.71 (0.55–1.00) vs. 0.54 (0.35–0.75) mm/mmHg, FAC/PASP 1.54 ± 0.61 vs. 1.24 ± 0.75%/mmHg, all *P* < 0.01]. RVFWS/PASP identified RV‐PA uncoupling was associated with a higher risk of all‐cause mortality (hazard ratio 4.64, 95% confidence interval 1.34–16.09, *P* = 0.033).

**Conclusions:**

Persistent RV dysfunction, as indicated by both standard and advanced echocardiographic markers during the early follow‐up period, implies a reduced potential for long‐term LV recovery in patients with newly diagnosed HFrEF.

## Introduction

Right ventricular (RV) dysfunction is an independent predictor of survival in patients with chronic heart failure (HF) that forecasts poor outcomes more accurately than systolic left ventricular (LV) dysfunction.^1^, ^2^, ^3^ RV dysfunction can occur as a consequence of systemic involvement in HF or as a secondary response to elevated pulmonary pressures in advanced disease stages. In addition to conventional echocardiographic measures, there has been an increasing interest to analyse the predictive value of advanced RV function parameters. RV global longitudinal strain (RVGLS) and RV free wall longitudinal strain (RVFWS) are both known to be associated with outcomes in chronic HF.^4^, ^5^ RVFWS provided additional prognostic value when compared to classical markers of RV function.^4^ Furthermore, strain analysis‐based indices of RV myocardial work were prognostic and predicted early RV failure in patients undergoing LVAD implantation.^6^ RV‐pulmonary artery (PA) coupling serves as parameter of RV function adjusted for afterload. While invasive measurements using pressure**–**volume loops provide the most reliable results, recent attention has focused on non‐invasive echocardiographic methods. The ratios of tricuspid annular plane systolic excursion (TAPSE) to pulmonary artery systolic pressure (PASP) and RV longitudinal strain to PASP both predicted survival and HF hospitalization rates irrespective of the LV ejection fraction (LVEF).^7^, ^8^

Early identification of myocardial recovery potential is crucial for the management of device therapy in newly diagnosed HFrEF. The current guidelines recommend considering an implantable cardioverter‐defibrillator (ICD) for primary prevention in patients with an LVEF ≤35% after 3 months of guideline‐recommended medical therapy (GDMT).^9^ However, in some cases, the systolic LV function may improve to the extent that an ICD may become unnecessary after a period longer than 3 months, particularly when GDMT has still not been fully uptitrated.^10^

Currently, there is insufficient evidence regarding predictors of myocardial recovery. The early identification of HFrEF patients with LV improvement potential, who would benefit from a strategy of postponing ICD implantation, holds significant clinical importance. It is unknown whether the advanced parameters of RV function could be useful in assessing the potential for LV improvement. Considering that RV dysfunction might represent the endpoint of HFrEF, we hypothesized that ongoing RV dysfunction could serve as a predictor for the potential improvement in LV function. Therefore, this study aimed to evaluate whether the RV function at early follow‐up can predict long‐term LVEF improvement in patients with newly diagnosed HFrEF.

## Methods

### Data source and study design

This is a retrospective sub‐analysis of patients included in the PROLONG‐II study. The PROLONG‐II study was an observational single‐centre study that analysed patients with new onset HFrEF receiving a wearable cardioverter‐defibrillator (WCD) (LifeVest®, Zoll Medical Corp., Pittsburgh, PA, USA) for primary prevention of sudden cardiac death (SCD) at our institution between 2012 and 2017. The details of study's rationale, design, and results have been reported elsewhere.^11^ Briefly, the long‐term prognosis of patients with newly diagnosed HFrEF receiving a WCD was analysed.

To test if advanced parameters of RV function can be used to assess the potential for LVEF improvement in long term in patients with newly diagnosed HFrEF, patients were included in this analysis if they received echocardiographic follow‐up assessments at our site. Early follow‐up was defined as the echocardiographic assessment 3 months after the onset of HFrEF, while the last follow‐up was defined as the most recent available assessment. Patients in whom echocardiography data were not available or those with insufficient quality for the extended evaluation of RV function were excluded. Advanced parameters of systolic RV function were analysed at two time points: baseline (diagnosis of HFrEF and prescription of WCD) and early (3‐month) follow‐up. HFrEF was defined as LVEF ≤40%. For the primary analysis, patients were divided into two subgroups based on their LVEF, which serves as established threshold for the indication for ICD therapy, at the last available long‐term follow‐up: the LVEF improvement subgroup (LVEF >35%) and the LVEF non‐improvement subgroup (LVEF ≤35%). The investigation conforms with the principles outlined in the Declaration of Helsinki and received approval from the local ethics committee. All study participants provided written informed consent.

### Echocardiography and speckle‐tracking strain analysis

Transthoracic echocardiography was performed using Philips iE33, EPIQ 7, or EPIQ CVx (Philips Electronics, Eindhoven, The Netherlands). All echocardiographic measurements were performed in accordance with the guidelines.^12^ LVEF was calculated using Simpson's biplane method from apical two‐ and four‐chamber views. The following RV parameters were analysed: RVFWS (performed offline), TAPSE, fractional area change (FAC, performed offline), maximal tricuspid regurgitation velocity (TRV_max_), estimated PASP, and RV end‐diastolic diameter (RVEDD). TAPSE was measured within M‐mode intersecting the lateral tricuspid annulus. FAC was measured offline by tracing the RV endocardial borders at end‐diastole and end‐systole in the apical four‐chamber view with focus on RV. PASP was estimated by measurement of TRV_max_ and right atrial pressure (RAP): PASP = 4(TRV_max_)^2^ + RAP. RAP was estimated from the inferior vena cava diameter and change with inspiration.

The TOMTEC Image Arena TTA 2.21.03/ISCV‐TOMTEC‐Integration TTA2.41.00 software (TOMTEC Imaging Systems GMBH, Unterschleissheim, Germany) was used for the offline speckle‐tracking strain and FAC analysis. The peak longitudinal systolic strain of the RV free wall and interventricular septum was measured from an apical four‐chamber view with focus on RV. Strain analysis was performed in patients with adequate RV endomyocardial border definition that was traced automatically by the software after setting the reference points and adapted manually for the optimal tracking throughout the cardiac cycle. The RVFWS is automatically divided by the software into basal, middle, and apical segments. The averaged, software‐generated mean value of RVFWS was used for the analysis.

RVFWS, TAPSE, and FAC were classified according to clinically significant thresholds, indicative of RV dysfunction: RVFWS > −23%, TAPSE < 17 mm, and FAC < 35%.^12^ The ratios of RVFWS/PASP, TAPSE/PASP, and FAC/PASP were used to assess the right ventricular‐pulmonary artery (RV‐PA) coupling. Median values were used to identify RV‐PA uncoupling.

### Statistical analysis

Continuous variables are demonstrated as mean ± standard deviation or as median [interquartile range (IQR)] depending on the presence of a normal distribution, and categorical variables as counts and percentages. Continuous data were evaluated for normality of distribution with the Shapiro–Wilk test. The two‐sided *t*‐test was used for comparison of continuous, normally distributed data, the non‐parametric Mann–Whitney U‐test for non‐normally distributed data. Associations between categorical variables were tested using the chi‐square (*x* ^2^) test. Survival analysis was performed by the Kaplan–Meier method. *P* values <0.05 were considered statistically significant. Univariable and multivariable logistic regression analysis was used to examine the association of clinical variables with LVEF improvement >35%. Variables suggesting significant differences with regard to later LV improvement were included in the univariable regression analysis. Variables showing significant results in the univariable regression analysis were included in the multivariable model. Due to the fact of multicollinearity, of the RV parameters, only RVWFS was tested in univariable and multivariable analysis due to its robustness, low investigator dependence, and proven superior prognostic value. Analyses were performed using SPSS version 29 (IBM Corp., Armonk, NY) and GraphPad Prism version 10 (GraphPad Software, San Diego, CA).

## Results

### Study population

The PROLONG‐II trial included 353 patients with newly diagnosed HFrEF. A total of 93 patients (26%) were excluded from the present analysis: 87 patients were excluded due to inadequate tracking of the RV endomyocardial border for speckle‐strain analysis or dropout of the RV free wall segment, one patient died before the scheduled follow‐up, and in five cases, follow‐up echocardiography was not available. Consequently, the final analytic cohort comprised 260 patients. For further investigation of advanced RV function parameters, only patients with available RV strain measures at both baseline and early follow‐up were included in the analysis (*n* = 215). The derivation of analytic study cohort is demonstrated in *Figure* *S1*.

### Baseline characteristics

The mean age was 57 ± 15 years, and 68% were male. The majority of patients (57%) had a New York Heart Association (NYHA) class III or IV and had severely reduced systolic LV function (LVEF 24.0 ± 6.9%). Measures of RV function (mean RVFWS −16.3%, TAPSE 16.0 mm, and FAC 32.3%) revealed reduced RV function at baseline, as well as derangement in RV‐PA coupling. Non‐ischaemic cardiomyopathies were the main cause of HF (63%). The study patients were on optimal GDMT: 94% received beta‐blockers, 97% renin–angiotensin–aldosterone system inhibitors, and 90% mineralocorticoid receptor antagonists [sodium‐glucose cotransporter‐2 (SGLT2) inhibitors were not approved for HF at the time of study inclusion]. However, given the early phase at baseline, the target dose had not yet been reached. Baseline characteristics of the study patients are summarized in *Table* *1*.

**Table 1:** Baseline characteristics of the study population

	All patients *n* = 260	LVEF improvement *n* = 151	LVEF non‐improvement *n* = 109	*P*
Demographic and medical history data				
Age, years	57.0 ± 15.0	53.6 ± 15.0	61.9 ± 13.7	<0.001
Male sex, *n* (%)	176 (68)	91 (60)	85 (78)	0.003
NYHA class, *n* (%)				
I	6 (2)	2 (1)	4 (4)	0.207
II	108 (42)	62 (41)	46 (42)	
III	111 (43)	63 (42)	48 (44)	
IV	35 (14)	24 (16)	11 (10)	
Aetiology of heart failure, *n* (%)				
Ischaemic heart disease	95 (37)	36 (24)	59 (54)	<0.001
Significant RCA stenosis or occlusion, *n* (%)	47 (18)	17 (11)	30 (28)	<0.001
Dilated cardiomyopathy	121 (47)	78 (52)	43 (39)	0.052
Peripartum cardiomyopathy	20 (8)	19 (13)	1 (1)	<0.001
Myocarditis	17 (7)	14 (9)	3 (3)	0.042
Other	7 (3)	4 (3)	3 (3)	1.000
Comorbidities, *n* (%)				
Hypertension	142 (55)	73 (48)	69 (63)	0.017
Diabetes mellitus	61 (24)	24 (16)	37 (34)	<0.001
Dyslipidaemia	88 (34)	43 (29)	45 (41)	0.031
Chronic kidney disease	60 (23)	27 (18)	33 (30)	0.019
Chronic obstructive pulmonary disease	19 (7)	11 (7)	8 (7)	0.970
Obstructive sleep apnoea	10 (4)	3 (2)	7 (4)	0.099
Medications				
Beta‐blockers, *n* (%)	245 (94)	144 (95)	101 (93)	0.356
% of target beta‐blocker dose	47.8 ± 26.7	48.5 ± 26.8	46.8 ± 26.8	0.609
Reached target dose of beta‐blockers, *n* (%)	37 (14)	22 (15)	15 (14)	0.854
ACEi/ARB/ARNI, *n* (%)	252 (97)	148 (98)	104 (95)	0.231
% of target ACEi/ARB/ARNI dose	46.9 ± 26.2	46.2 ± 25.7	47.9 ± 27.0	0.603
Reached target dose of ACEi/ARB/ARNI, *n* (%)	31 (12)	17 (11)	14 (13)	0.697
MRA, *n* (%)	233 (90)	138 (91)	95 (87)	0.269
% of target MRA dose	40.1 ± 22.1	48.5 ± 19.8	49.8 ± 24.9	0.645
Reached target dose of MRA, *n* (%)	25 (10)	11 (7)	14 (13)	0.134
Loop diuretics, *n* (%)	209 (80)	124 (82)	85 (78)	0.407
Ivabradine, *n* (%)	55 (21)	39 (26)	16 (15)	0.027
Digitalis, *n* (%)	23 (9)	12 (8)	11 (10)	0.548
LVEF at baseline, %	24.0 ± 6.9	24.5 ± 7.1	23.2 ± 6.6	0.143
NT‐proBNP (pg/mL)	3697 (1249–7380)	3187 (840–6805)	4205 (1624–8058)	0.201

ACEi, angiotensin‐converting enzyme inhibitor; ARB, angiotensin (II) receptor blocker; ARNI, angiotensin receptor‐neprilysin inhibitor; LVEF, left ventricular ejection fraction; MRA, mineralocorticoid receptor antagonists; NT‐proBNP, N‐terminal pro B‐type natriuretic peptide; NYHA, New York Heart Association.

### Clinical features associated with long‐term left ventricular ejection fraction improvement

One hundred fifty‐one (58%) patients experienced LV improvement in the long term during a median follow‐up period of 31.5 months (IQR: 18.2–45.4). Among them, 69 (46%) had an LVEF ≤35% at early follow‐up. The subgroup of study patients with long‐term LVEF improvement were younger (53.6 vs. 61.9 years, *P* < 0.001), and the proportion of males was lower (60 vs. 78%, *P* = 0.003). Patients with non‐ischaemic HF aetiology were more likely to experience long‐term LV improvement (dilated cardiomyopathy as cause of HFrEF in 52 vs. 39%, *P* = 0.052; ischaemic heart disease in 24 vs. 54%, *P* < 0.001; peripartum cardiomyopathy in 13 vs. 1%, *P* < 0.001; myocarditis in 9 vs. 3%, *P* = 0.042). Similarly, the rates of long‐term LVEF improvement were lower in those with significant right coronary artery (RCA) stenosis or occlusion. On the other hand, in the subgroup analysis of patients with ischaemic HF origin, there were no differences regarding RCA involvement between those with and without long‐term LVEF improvement (47% vs. 46%, respectively, *P* = 0.890). No differences were observed between the groups regarding NYHA functional class, LVEF, and N‐terminal pro B‐type natriuretic peptide (NT‐proBNP) levels at baseline (*Table* *1*). Patients in the LV non‐improvement group carried a higher burden of comorbidities (all *P* < 0.05).

### Right ventricular function and right ventricular‐pulmonary artery coupling at early follow‐up as marker for long‐term left ventricular improvement

RVFWS revealed the highest rates of RV dysfunction (74%) during the early follow‐up assessment, compared to TAPSE (32%) and FAC (34%). No differences were observed in the standard and advanced parameters of RV function at baseline between the LVEF improvement and non‐improvement groups (*Table* *2*). However, significant changes were evident during the early follow‐up period. The patient subgroup with improved long‐term LVEF had lower rates of RV dysfunction at early follow‐up compared to non‐improvers (67 vs. 85%, *P* = 0.005 by RVFWS, 26 vs. 42%, *P* = 0.033 by TAPSE, and 24 vs. 49%, *P* < 0.001 by FAC). Furthermore, the change in all analysed RV variables between baseline and early follow‐up was higher in the LV recovery subgroup [3.5% (IQR: 0.8–7.6) vs. 2.2% (IQR: −0.17–5.6), *P* = 0.022 for RVFWS, 3.9 vs. 1.7 mm, *P* = 0.022 for TAPSE, and 5.1% (IQR: 0.9–13.1) vs. 2.5% (IQR: −0.9–7.2), *P* = 0.026 for FAC]. Moreover, patients without LV improvement had significantly lower markers of RV‐PA coupling at early follow‐up, whereas there were no statistically significant differences at baseline (*Table* *3*).

**Table 2:** RV systolic function in patients with and without long‐term LVEF improvement

	All patients *n* = 215	LVEF improvement *n* = 131	LVEF non‐improvement *n* = 84	*P*
RVFWS at baseline, %	−16.3 ± 5.4	−16.6 ± 5.4	−15.9 ± 5.3	0.355
RVFWS at early follow‐up, %	−20.0 ± 4.8	−20.9 ± 4.3	−18.5 ± 5.1	<0.001
Delta‐RVFWS, %	3.2 (0.4–6.9)	3.5 (0.8–7.6)	2.2 (−0.17–5.6)	0.022
RVFWS identified RV dysfunction at early follow‐up^a^, *n* (%)	159 (74)	88 (67)	71 (85)	0.005
TAPSE at baseline, mm	16.0 ± 5.0	16.2 ± 4.8	15.6 ± 5.3	0.449
TAPSE at early follow‐up, mm	18.8 ± 5.1	19.7 ± 5.1	17.4 ± 4.9	0.002
Delta‐TAPSE, mm	3.0 ± 5.6	3.9 ± 5.8	1.7 ± 5.1	0.022
TAPSE identified RV dysfunction at early follow‐up^a^, *n* (%)	53 (32)	26 (26)	27 (42)	0.033
FAC at baseline, %	32.3 ± 10.5	33.1 ± 10.7	31.2 ± 10.2	0.205
FAC at early follow‐up, %	38.0 ± 9.1	39.7 ± 8.5	35.2 ± 9.4	<0.001
Delta‐FAC, %	3.6 (0.2–10.3)	5.1 (0.9–13.1)	2.5 (−0.9–7.2)	0.026
FAC identified RV dysfunction at early follow‐up^a^, *n* (%)	72 (34)	32 (24)	40 (49)	<0.001
PASP at baseline, mmHg	36 (25–45)	35 (25–45)	38 (25–50)	0.636
PASP at early follow‐up, mmHg	30 (23–36)	26 (22–35)	31 (25–46)	0.018
Delta‐PASP, mmHg	5 (−1–13)	6 (1–16)	3 (−5–15)	0.078

FAC, fractional area change; LVEF, left ventricular ejection fraction; PASP, pulmonary artery systolic pressure; RV, right ventricle/right ventricular; RVFWS, right ventricular free wall longitudinal strain; TAPSE, tricuspid annular plane systolic excursion.

At early follow‐up refers to the echocardiographic assessment conducted 3 months after the onset of HFrEF.

^a^ Thresholds indicating RV dysfunction: RVFWS < −23%, TAPSE < 17 mm, FAC < 35%.

**Table 3:** RV‐PA coupling in patients with and without long‐term LVEF improvement

Variable	All patients *n* = 215	LVEF improvement *n* = 131	LVEF non‐improvement *n* = 84	*P*
TAPSE/PASP at baseline, mm/mmHg	0.40 (0.29–0.56)	0.40 (0.29–0.61)	0.41 (0.29–0.55)	0.511
TAPSE/PASP at early follow‐up, mm/mmHg	0.67 (0.41–0.88)	0.71 (0.55–1.00)	0.54 (0.35–0.75)	0.002
Delta‐TAPSE/PASP	0.22 ± 0.34	0.28 ± 0.30	0.11 ± 0.38	0.018
TAPSE/PASP identified RV‐PA uncoupling at early follow‐up^a^, *n* (%)	57 (51)	29 (43)	28 (65)	0.021
FAC/PASP at baseline, %/mmHg	0.83 (0.54–1.26)	0.83 (0.54–1.35)	0.83 (0.56–1.10)	0.398
FAC/PASP at early follow‐up, %/mmHg	1.43 ± 0.68	1.54 ± 0.61	1.24 ± 0.75	0.018
Delta‐FAC/PASP	0.43 ± 0.59	0.54 ± 0.55	0.24 ± 0.63	0.014
FAC/PASP identified RV‐PA uncoupling at early follow‐up^a^, *n* (%)	62 (50)	30 (39)	32 (64)	0.002
RVFWS/PASP at baseline, %/mmHg	0.45 (0.29–0.63)	0.43 (0.29–0.66)	0.45 (0.28–0.62)	0.626
RVFWS/PASP at early follow‐up, %/mmHg	0.75 ± 0.36	0.82 ± 0.35	0.65 ± 0.35	0.008
Delta‐RVFWS/PASP	0.26 ± 0.31	0.33 ± 0.30	0.14 ± 0.30	0.002
RVFWS/PASP identified RV‐PA uncoupling at early follow‐up^a^, *n* (%)	61 (49)	32 (42)	29 (59)	0.062

FAC/PASP, ratio between fractional area change and pulmonary artery systolic pressure; LVEF, left ventricular ejection fraction; RVFWS/PASP, ratio between right ventricular free wall longitudinal strain and pulmonary artery systolic pressure; RV‐PA, right ventricular‐pulmonary artery; TAPSE/PASP, ratio between tricuspid annular plane systolic excursion ratio and pulmonary artery systolic pressure.

At early follow‐up refers to the echocardiographic assessment conducted 3 months after the onset of HFrEF.

^a^ Thresholds indicating RV‐PA uncoupling: RVFWS/PASP ≤ 0.71%/mmHg, TAPSE/PASP ≤ 0.67 mm/mmHg, FAC/PASP ≤ 1.41%/mmHg.

The analysis of the patient subgroup with reduced 3‐month LVEF similarly demonstrated worse RV function at the early follow‐up in non‐improvers with no differences at baseline assessment (*Table* *S2*).

In multivariable analysis, younger age and better RVFWS at early follow‐up proved to be independent predictors of LVEF improvement (*Table* *4*). All markers of systolic RV function at early follow‐up but not at baseline were predictive of later LV improvement in univariable analysis (*Table* *S3*). Due to multicollinearity, only RVWFS was tested in multivariable analysis.

**Table 4:** Predictors of long‐term LVEF improvement (LVEF > 35% at latest follow‐up)

	Univariable analysis		Multivariable analysis	
	OR (95% CI)	*P*	OR (95% CI)	*P*
Age	0.960 (0.941–0.980)	<0.001	0.976 (0.954–0.998)	0.032
Female sex	2.852 (1.511–5.384)	0.001	1.748 (0.863–3.541)	0.121
NICM	3.197 (1.764–5.794)	<0.001	1.588 (0.779–3.236)	0.203
Hypertension	0.575 (0.330–1.001)	0.050		
Diabetes mellitus	0.324 (0.169–0.623)	0.001	0.562 (0.265–1.194)	0.134
Dyslipidaemia	0.648 (0.361–1.164)	0.147		
Chronic kidney disease	0.619 (0.327–1.173)	0.141		
RVFWS at early follow‐up	1.116 (1.050–1.186)	<0.001	1.078 (1.010–1.150)	0.024

95% CI, 95% confidence interval; LVEF, left ventricular ejection fraction; NICM, non‐ischaemic cardiomyopathy; OR, odds ratio; RVFWS, right ventricular free wall longitudinal strain.

At early follow‐up refers to the echocardiographic assessment conducted 3 months after the onset of HFrEF.

**Figure 1:**
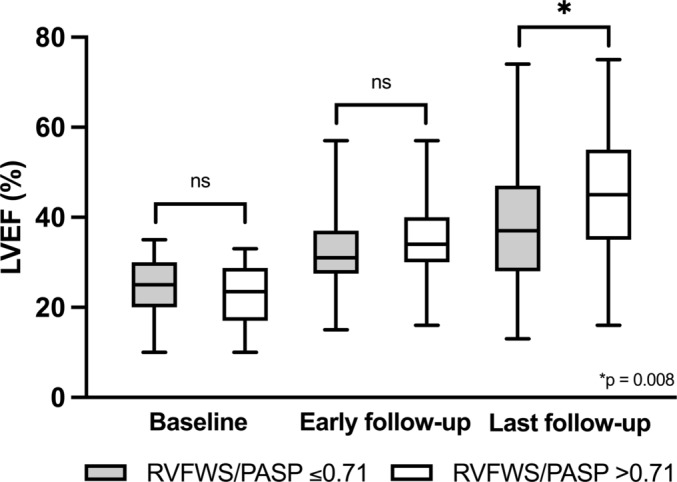
LVEF changes in subgroups with and without RV‐PA uncoupling as evaluated by RVFWS/PASP at baseline, early 3‐month, and last available follow‐up. LVEF, left ventricular ejection fraction; RV, right ventricular, RVFWS/PASP, ratio between right ventricular free wall longitudinal strain and pulmonary artery systolic pressure.

*Figure* *1* demonstrates the changes in LVEF within subgroups with and without RV‐PA uncoupling, as assessed by RVFWS/PASP at baseline, during early and long‐term follow‐up.

### All‐cause mortality by follow‐up right ventricular‐pulmonary artery uncoupling

**Figure 2:**
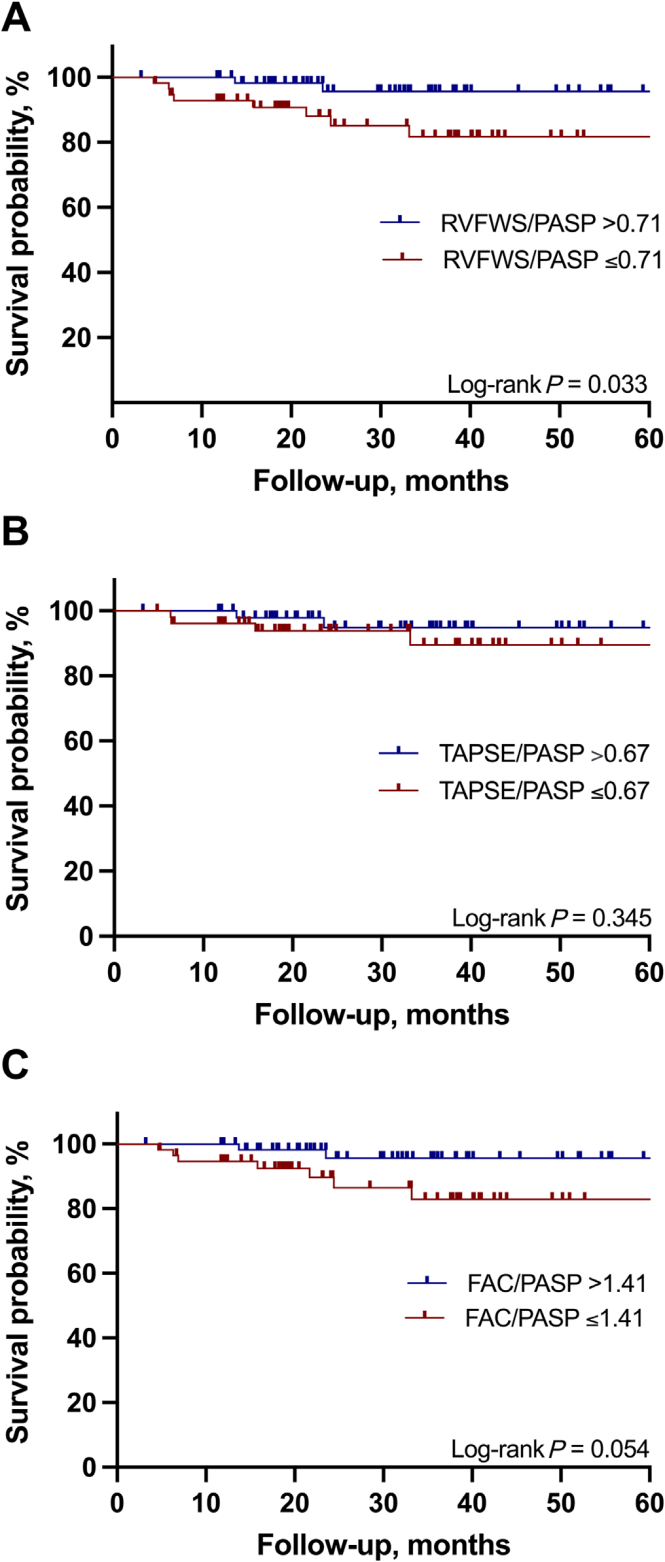
Kaplan–Meier survival curves according to RV‐PA coupling. (A) Survival analysis according to RVFWS/PASP (cut‐offs >0.71 vs. ≤0.71%/mmHg). (B) Survival analysis according to TAPSE/PASP (cut‐offs ≥0.67 vs. <0.67 mm/mmHg). (C) Survival analysis according to FAC/PASP (cut‐offs >1.41 vs. ≤1.41%/mmHg). FAC/PASP, ratio between fractional area change and pulmonary artery systolic pressure; RVFWS/PASP, ratio between right ventricular free wall longitudinal strain and pulmonary artery systolic pressure; RV‐PA, right ventricular‐pulmonary artery; TAPSE/PASP, ratio between tricuspid annular plane systolic excursion ratio and pulmonary artery systolic pressure.

During a median follow‐up of 31.5 months (IQR: 18.2–45.4), a total of 19 patients died, and six patients underwent implantation of a left ventricular assist device (LVAD), three patients underwent heart transplantation, and one patient was bridged to transplantation with a LVAD (all 10 LVAD/transplant patients were in the non‐recovery group). Kaplan–Meier estimates were constructed to analyse the mortality differences in patients with vs. without RV‐PA uncoupling (*Figure* *2*). The cut‐off of 0.71%/mmHg for RVFWS/PASP at early follow‐up was associated with a higher risk of all‐cause mortality (HR 4.64, 95% CI 1.34–16.09, *P* = 0.033). RVFWS identified RV dysfunction (>−23%) was not linked to survival difference (log‐rank *P* = 0.308). The cut‐offs of 0.67 mm/mmHg for TAPSE/PASP and 1.41%/mmHg for FAC/PASP did not exhibit a significant association with mortality.

## Discussion

This study is the first to investigate the predictive value of advanced echocardiographic RV function and RV‐PA coupling parameters in forecasting myocardial recovery in patients newly diagnosed with HFrEF. The results of this study demonstrate that (i) long‐term improvement in LVEF was associated with non‐ischaemic causes of HF, younger age, and female sex; (ii) persistent RV dysfunction during early follow‐up is associated with less long‐term changes in LVEF and can be used to predict recovery potential; and (iii) a RVFWS/PASP ratio of ≤0.71%/mmHg at early follow‐up predicts all‐cause mortality in patients with de novo HFrEF.

Recognizing myocardial recovery potential in patients with *de novo* HFrEF is crucial for optimizing device therapy management. For symptomatic HFrEF patients with a LVEF of ≤35% after at least three months of GDMT, an ICD is recommended for primary prevention of SCD.^9^ During this early period, a WCD serves as a temporary protective measure, providing a bridge during drug titration before considering ICD placement.^11^, ^13^, ^14^ This approach is particularly relevant for a subset of patients whose systolic LV function may improve to the extent that an ICD may no longer be necessary.^15^ However, due to the complexity of LV remodelling, improvements in LVEF might take longer than 3 months to manifest, and it remains unclear how long one should wait and in which patient subgroup LVEF improvement is most likely to occur.

### Early identification of reverse remodelling potential in heart failure with reduced ejection fraction

The process of reverse remodelling refers to the change of cardiac myocyte size and LV chamber geometry towards normal values and is linked to improved cardiac function and prognosis.^16^, ^17^ These changes typically occur in the context of GDMT/device therapies including LVAD, coronary revascularization, and valvular repair, which can result in partial or complete normalization of LVEF.^18^, ^19^ In our study, we defined LV improvement as LVEF greater than 35% as our primary objective was to assess the potential of early RV dysfunction in discriminating HFrEF patients who may not achieve LV recovery beyond the LVEF threshold for primary SCD prevention ICD therapy.

Several clinical, biomarker, and imaging parameters have been identified as predictors of reverse remodelling in patients with HFrEF. Consistent with the findings reported in previous trials,^20^, ^21^ non‐ischaemic cause of HF, younger age, and female sex were associated with long‐term LVEF improvement in our patient population. Furthermore, patients with certain types of non‐ischaemic cardiomyopathy are more likely to exhibit myocardial recovery.^21^ Similarly, peripartum cardiomyopathy and myocarditis were more prevalent among the subgroup of patients who demonstrated long‐term improvement in LVEF, even though showing an increased risk for life‐threatening arrhythmias in the early phase.^14^, ^22^, ^23^ In addition to classic natriuretic peptides, novel markers of HF and fibrosis such as galectin‐3,^24^ soluble suppression of tumorigenesis‐2,^25^ and serum collagen type I‐derived peptides^26^ have also emerged as independent predictors of myocardial recovery. Due to the fact that NT‐proBNP failed to distinguish between patients with and without long‐term LVEF improvement in our study, findings of this study emphasize the necessity of novel biomarkers in predicting the long‐term course of HFrEF. Different cardiac imaging techniques have evaluated the potential for cardiac remodelling. The absence of late gadolinium enhancement (LGE) in cardiac magnetic resonance imaging (CMR) in patients with dilated cardiomyopathy was found to predict reverse remodelling and a better prognosis, irrespective of baseline LV volumes and LVEF.^27^ Nonetheless, the limited availability and high costs of CMR restrict its use in predicting LV improvement, thus establishing echocardiography as the gold standard for assessing the potential for myocardial recovery, stratifying risk, and guiding both medical and device therapies in patients with HF.

### Right ventricular function for prediction of left ventricular improvement

The current findings provide insights into the predictive value of early RV dysfunction in discrimination between HFrEF patients with and without LVEF improvement potential. It has been shown that systolic RV dysfunction serves as an independent prognostic factor for outcomes across various LVEF ranges.^28^ Speckle‐tracking echocardiography has been increasingly used for the evaluation of RV function and may add important prognostic information. RVGLS and RVFWS both unmasked RV dysfunction despite normal classical RV function measures in patients with HFrEF.^29^ A combined evaluation of FAC and RVFWS improved risk stratification in patients with HFrEF due to dilated cardiomyopathy.^30^ Furthermore, in a study that compared the prognostic value of CMR and speckle‐tracking echocardiography, RVGLS was associated with better prediction of cardiovascular and all‐cause mortality as compared to TAPSE, FAC, and CMR‐derived RV ejection fraction and RVGLS.^4^ Our results corroborate these findings and provide new insights regarding the role of RV dysfunction in the early period after HFrEF onset. While baseline RV function did not differ between the groups with and without LVEF improvement, significant changes were evident during the early follow‐up period. This demonstrates that long‐term LV remodelling is a complex process involving the RV and its function. The findings of our study support the hypothesis that persistent RV dysfunction indicates an advanced disease stage and likely reflects a secondary response to elevated pulmonary pressures. The presence of RV dysfunction at early follow‐up was associated with long‐term LVEF changes. This finding could be valuable in the decision‐making process regarding device therapy in patients with HFrEF if our findings can be replicated in future studies.

### Right ventricular‐pulmonary artery coupling and prognostic value of right ventricular free wall longitudinal strain/systolic pulmonary artery pressure ratio

The RV‐PA coupling describes the adaptation of RV contractility to afterload and is ideally estimated invasively by calculating the ratio of RV end‐systolic elastance to PA elastance (Ees/Ea).^31^ After the initial interest to assess the prognostic value of RV‐PA coupling mostly in patients with pulmonary arterial hypertension, its use has been extended to various populations of HF patients. The RV‐PA coupling ratio Ees/Ea predicted medium‐term survival in patients with HFrEF and secondary pulmonary hypertension. Furthermore, severely reduced LV function was identified as independent predictor of prognostically unfavourable RV‐PA coupling.^32^

The invasive origin of measuring Ees/Ea catalysed the search for simple surrogate parameters. TAPSE/PASP ratio predicted all‐cause mortality in patients with HFrEF with and without secondary pulmonary hypertension although normalizing TAPSE for PASP did not further improve prognostic power.^33^ Similarly, FAC/PASP and RVFWS/PASP were independent outcome predictors in HFrEF secondary to dilated cardiomyopathy, but this also did not improve prognostic value.^30^ In the present study, RVFWS/PASP ratio of ≤0.71%/mmHg predicted all‐cause mortality, whereas neither TAPSE/PASP ratio of ≤0.67 mm/mmHg nor FAC/PASP ratio of ≤1.41%/mmHg was associated with survival in our patient population. Moreover, our findings highlight the role of load‐independent RV function parameters during the early HFrEF stage. While no survival differences were observed between patients with and without RVFWS identified RV dysfunction, load adjusted RVFWS was associated with poorer survival. Furthermore, RV‐PA uncoupling detected at the early follow‐up in patients with newly diagnosed HFrEF was associated with long‐term improvement in LVEF, which further extends the utility of RV‐PA coupling parameters.

In summary, we showed that RVFWS and parameters of RV‐PA coupling (RVFWS/PASP, TAPSE/PASP, and FAC/PASP), were associated with the long‐term potential for LVEF improvement. While both the standard and advanced markers of RV function were not different between patients with and without LVEF improvement at the first manifestation of HFrEF, the evaluation of RV function during the early follow‐up period unveiled the discriminative capacity of RV dysfunction in predicting the potential for LV recovery. Furthermore, we demonstrate the prognostic significance of RVFWS/PASP.

## Limitations

The study was conducted at a single centre, which restricts the generalizability of our findings. In addition to the standard parameters of RV function, we analysed the extended markers including RVFWS and RV‐PA coupling ratios, though there is no consensus on which RV strain parameter and RV‐PA ratio should be preferred in clinical practice. We used RVFWS because it demonstrated superiority in prediction of all‐cause mortality and hospitalizations related to HF compared to RVGLS.^5^ Unlike standard RV function parameters, there are no clear thresholds for the identification of RV‐PA uncoupling, and the thresholds used to detect this vary significantly across different HF patient cohort studies. This demonstrates a limitation of these parameters and underscores the need for the establishment of universal thresholds. Inter‐ and intraobserver variability is a possible limitation in this study. However, the impact is minimized since the measurements of RV function are part of the clinical routine and were made by experienced echocardiographers. Furthermore, automated software for strain assessment was used, which further reduced possible interobserver variability. Given that HF is a dynamic condition, analysing LV remodelling at specific time points rather than only at the last available follow‐up could help avoid significant bias. However, this was not feasible due to the retrospective study design, which highlights a limitation of our analysis.

## Conclusions

In patients with newly diagnosed HFrEF, the persistent RV dysfunction observed during the early follow‐up period indicates an advanced disease stage and suggests a lack of potential for LVEF improvement in the long term. Whereas the initial RV function did not predict the potential for LVEF recovery in our study, RV function at early follow‐up was associated with LV recovery in the long term. The ability to predict the potential for LV recovery may facilitate early management decisions regarding device therapy in patients with HFrEF.

## Conflict of interest

H.A.K.H. received modest lecture honorary and/or a fellowship grant from AstraZeneca, Zoll, and Boston Scientific. J.M. received modest lecture honorary, travel grants, and/or a fellowship grant from Abbott, Biotronik, Boston Scientific, and Zoll. J.E. received a fellowship grant from Biotronik. C.V. received honorary for lectures or consulting from Abbott, Medtronic, and Zoll. J.B. received honoraria for lectures/consulting from Novartis, Vifor, Bayer, Pfizer, Boehringer Ingelheim, AstraZeneca, Cardior, CVRx, BMS, Amgen, Corvia, Norgine, Edwards, and Roche not related to this article; and research support for the department from Zoll, CVRx, Abiomed, Norgine, and Roche, not related to this article. D.D. received modest lecture honorary, travel grants, and/or a fellowship grant from Abbott, AstraZeneca, Biotronik, Boehringer Ingelheim, Boston Scientific, Bristol Myers Squibb, CVRx, Medtronic, Microport, Pfizer, Sanofi, and Zoll. D.B. received honoraria for lectures/consulting from Abbott Vascular, AstraZeneca, Boehringer Ingelheim, Bristol‐Myers Squibb, Daiichi Sankyo, Edwards Lifesciences, and Pfizer. All other authors: none declared.

## Funding

D.B. was competitively selected for ‘CORE100Pilot’, which is an advanced clinician–scientist programme co‐funded by the Else Kroner‐Fresenius Foundation and the Lower Saxony Ministry of Science and Culture.

## Supporting information


**Figure S1.** Study flow chart.

**Table S1.** Changes of systolic left ventricular Function of the study population.
**Table S2.** RV systolic function and RV‐PA coupling in the subgroup of patients with reduced LVEF at 3 months.
**Table S3.** Univariable analysis of markers of RV systolic function for the prediction of long‐term LVEF improvement.
