# Supplementary figures and images for: Right ventricular dysfunction for prediction of long‐term recovery in de novo HFrEF : a PROLONG‐II substudy
Source: ESC Heart Fail. 2025 Feb 4;12(3):2166–76. doi: 10.1002/ehf2.15236 (PMC12055338; doi:10.1002/ehf2.15236)

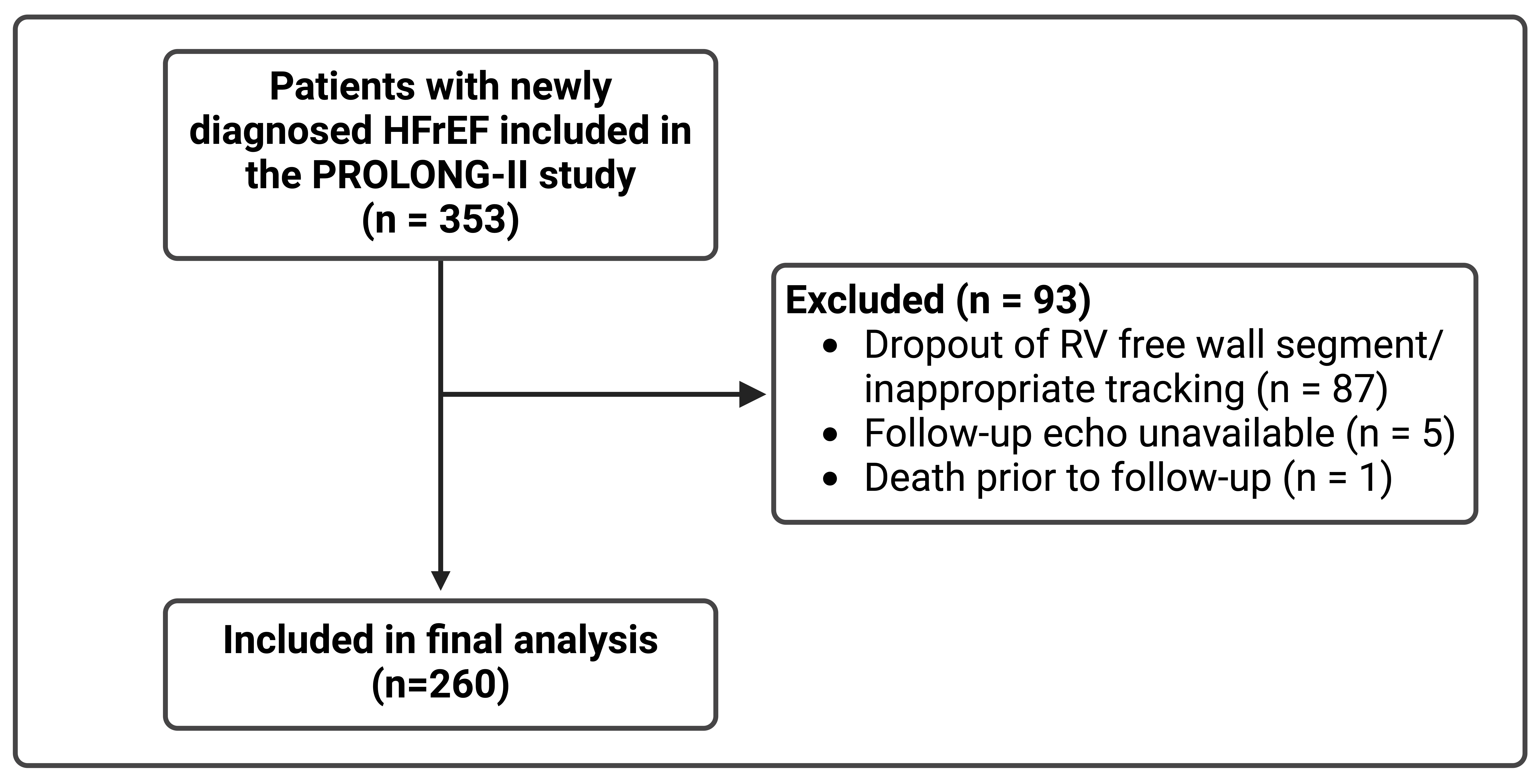

Supplement: Supplementary file 1 — Figure S1. Study flow chart. [file EHF2-12-2166-s001.png]
