# Supplementary material for: Right ventricular dysfunction for prediction of long‐term recovery in de novo HFrEF : a PROLONG‐II substudy
Source: ESC Heart Fail. 2025 Feb 4;12(3):2166–76. doi: 10.1002/ehf2.15236 (PMC12055338; doi:10.1002/ehf2.15236)
Supplement: Supplementary file 2 — Table S1. Changes of systolic left ventricular Function of the study population. Table S2. RV systolic function and RV‐PA coupling in the subgroup of patients with reduced LVEF at 3 months. Table S3. Univariable analysis of markers of RV systolic function for the prediction of long‐term LVEF improvement. [file EHF2-12-2166-s002.docx]

**Appendices**

**Supplementary Figures**

**Figure S1.** Study flow chart


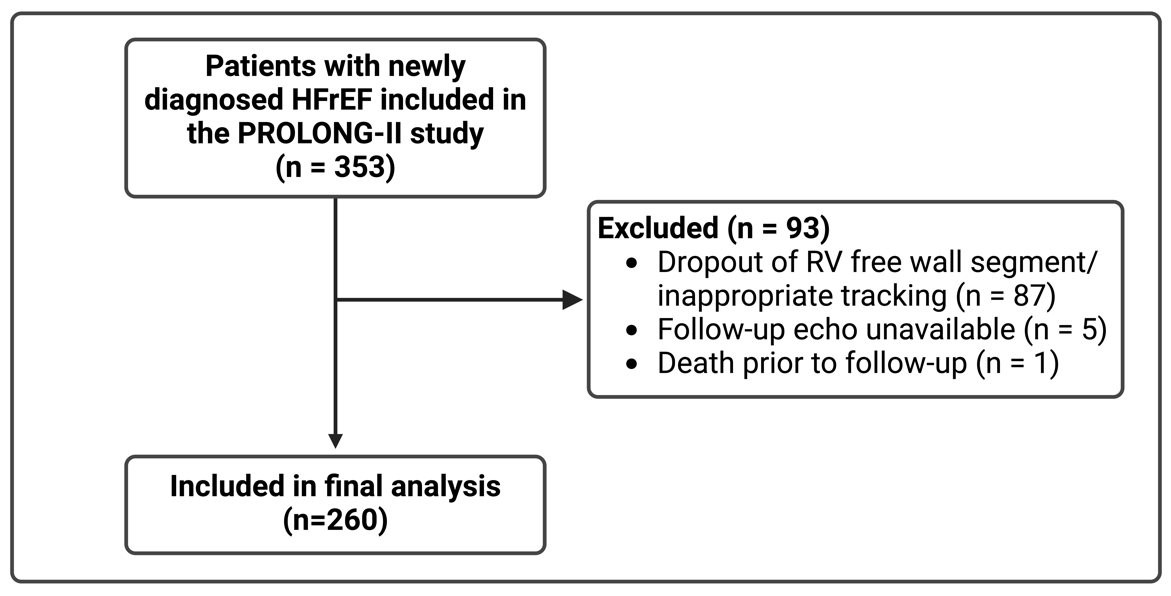


HFrEF = heart failure with reduced ejection fraction, RV = right ventricle.

**Supplementary tables**

**Table S1.** Changes of systolic left ventricular Function of the study population

|  | All patients  n=260 | LVEF improvement  n=151 | LVEF  non-improvement  n=109 | p |
| --- | --- | --- | --- | --- |
| LVEF at baseline, % | 24.0 ± 6.9 | 24.5 ± 7.1 | 23.2 ± 6.6 | 0.143 |
| LVEF at early follow-up, % | 33.3 ± 9.5 | 37.3 ± 8.8 | 27.4 ± 7.0 | <0.001 |
| Delta-LVEF, % | 9.4 ± 10.2 | 12.8 ± 10.0 | 4.4 ± 8.1 | <0.001 |
| LVGLS at baseline, % | -7.9 ± 3.1 | -8.2 ± 3.1 | -7.4 ± 3.0 | 0.072 |
| LVGLS at early follow-up, % | -11.7 ± 3.9 | -13.3 ± 3.4 | -8.9 ± 3.2 | <0.001 |
| Delta-LVGLS, % | -4.0 ± 3.9 | -5.4 ± 3.9 | -1.7 ± 2.6 | <0.001 |

LVGLS = left ventricular global longitudinal strain, LVEF = left ventricular ejection fraction

**Table S2.** RV systolic function and RV-PA coupling in the subgroup of patients with reduced LVEF at 3 months

|  | Subgroup with reduced LVEF at 3-month follow-up  n=136 | LVEF improvement  n=62 | LVEF non-improvement  n=74 | p |
| --- | --- | --- | --- | --- |
| RVFWS at baseline, % | -15.72 ± 5.45 | -15.62 ± 5.38 | -15.81 ± 5.54 | 0.837 |
| RVFWS at early follow-up, % | -19.30 ± 4.96 | -20.34 ± 4.49 | -18.43 ± 5.19 | 0.025 |
| TAPSE at baseline, mm | 15.52 ± 5.16 | 15.60 ± 4.63 | 15.46 ± 5.59 | 0.887 |
| TAPSE at early follow-up, mm | 19 (14-22) | 20 (15-23) | 18 (13-21) | 0.100 |
| FAC at baseline, % | 32.10 ± 10.68 | 32.86 ± 10.95 | 31.45 ± 10.47 | 0.447 |
| FAC at early follow-up, % | 38 (29-45) | 40 (33-47) | 37 (25-43) | 0.039 |
| PASP at baseline, mmHg | 38 (25-44) | 36 (26-44) | 40 (25-45) | 0.792 |
| PASP at early follow-up, mmHg | 30 (25-38) | 30 (24-35) | 31 (25-44) | 0.168 |
| TAPSE/PASP at baseline, mm/mmHg | 0.40 (0.29-0.54) | 0.40 (0.31-0.52) | 0.40 (0.27-0.55) | 0.887 |
| TAPSE/PASP at early follow-up, mm/mmHg | 0.63 (0.38-0.80) | 0.71 (0.54-0.83) | 0.46 (0.34-0.77) | 0.065 |
| FAC/PASP at baseline, %/mmHg | 0.83 (0.53-1.12) | 0.82 (0.51-1.13) | 0.83 (0.56-1.11) | 0.962 |
| FAC/PASP at early follow-up, %/mmHg | 1.35 ± 0.72 | 1.47 ± 0.63 | 1.25 ± 0.80 | 0.192 |
| RVFWS/PASP at baseline, %/mmHg | 0.42 (0.27-0.56) | 0.39 (0.27-0.52) | 0.45 (0.27-0.62) | 0.339 |
| RVFWS/PASP at early follow-up, %/mmHg | 0.65 (0.40-0.90) | 0.73 (0.52-0.98) | 0.56 (0.37-0.84) | 0.060 |

FAC = fractional area change, LVEF = left ventricular ejection fraction, PASP = pulmonary artery systolic pressure, RVFWS = right ventricular free wall strain, RVFWS/PASP = right ventricular free wall strain to pulmonary artery systolic pressure ratio, TAPSE = tricuspid annular plane systolic excursion, TAPSE/PASP = tricuspid annular plane systolic excursion to pulmonary artery systolic pressure ratio, FAC/PASP = fractional area change to pulmonary artery systolic pressure ratio.

At early follow-up refers to the echocardiographic assessment conducted three months after the onset of HFrEF.

**Table S3**: Univariable analysis of markers of RV systolic function for the prediction of long-term LVEF improvement

|  | **Univariable analysis** | |
| --- | --- | --- |
|  | **OR (95%CI)** | **p** |
| **RVFWS at baseline (per -%)** | 1.025 (0.973-1.079) | 0.353 |
| **RVFWS at early follow-up (per -%)** | 1.116 (1.050-1.186) | <0.001 |
| **TAPSE at baseline (per mm)** | 1.024 (0.963-1.089) | 0.447 |
| **TAPSE at early follow-up (per mm)** | 1.097 (1.028-1.170) | 0.005 |
| **FAC at baseline (per %)** | 1.017 (0.991-1.045) | 0.204 |
| **FAC at early follow-up (per %)** | 1.059 (1.025-1.093) | 0.001 |
| **PASP at baseline (per mmHg)** | 0.993 (0.971-1.016) | 0.564 |
| **PASP at early follow-up (per mmHg)** | 0.958 (0.929-0.988) | 0.007 |
| **TAPSE/PASP at baseline (per mm/mmHg)** | 0.983 (0.308-3.137) | 0.977 |
| **TAPSE/PASP at early follow-up (per mm/mmHg)** | 5.752 (1.567-21.117) | 0.008 |
| **FAC/PASP at baseline (per %/mmHg)** | 1.102 (0.667-1.823) | 0.704 |
| **FAC/PASP at early follow-up (per %/mmHg)** | 2.044 (1.113-3.754) | 0.021 |
| **RVFWS/PASP at baseline (per %/mmHg)** | 1.150 (0.395-3.346) | 0.797 |
| **RVFWS/PASP at early follow-up (per %/mmHg)** | 4.505 (1.421-14.285) | 0.011 |

95%CI = 95% confidence interval, FAC = fractional area change, LVEF = left ventricular ejection fraction, OR = odds ratio, PASP = pulmonary artery systolic pressure, RV = right ventricle/right ventricular, RVFWS = right ventricular free wall longitudinal strain, RVFWS/PASP = right ventricular free wall strain to pulmonary artery systolic pressure ratio, TAPSE = tricuspid annular plane systolic excursion, TAPSE/PASP = tricuspid annular plane systolic excursion to pulmonary artery systolic pressure ratio, FAC/PASP = fractional area change to pulmonary artery systolic pressure ratio.

At early follow-up refers to the echocardiographic assessment conducted three months after the onset of HFrEF.
